# Discovery and Preclinical Evaluation of a Novel Inhibitor of FABP5, ART26.12, Effective in Oxaliplatin-Induced Peripheral Neuropathy

**DOI:** 10.1016/j.jpain.2024.01.335

**Published:** 2024-01-15

**Authors:** George Warren, Myles Osborn, Christopher Tsantoulas, Ana David-Pereira, Daniel Cohn, Paul Duffy, Linette Ruston, Clare Johnson, Heather Bradshaw, Martin Kaczocha, Iwao Ojima, Andrew Yates, Saoirse E O’Sullivan

**Affiliations:** * Artelo Biosciences Limited, UK; † Transpharmation Ltd., London, UK; ‡ Apconix Ltd., UK; § Seda Pharma Development Services Ltd., UK; ¶ Department of Psychological and Brain Sciences, Bloomington, Indiana; ‖ Department of Anesthesiology, Stony Brook University, New York; ** Department of Chemistry, Stony Brook University, New York; †† Institute of Chemical Biology and Drug Discovery, Stony Brook University, New York

**Keywords:** Chemotherapy-induced peripheral neuropathy, Fatty acid bind protein, Pain, Cannabinoid, Lipid

## Abstract

Oxaliplatin-induced peripheral neuropathy (OIPN) is a dose-limiting toxicity characterised by mechanical allodynia and thermal hyperalgesia, without any licensed medications. ART26.12 is a fatty acid-binding protein (FABP) 5 inhibitor with antinociceptive properties, characterised here for the prevention and treatment of OIPN. ART26.12 binds selectively to FABP5 compared to FABP3, FABP4, and FABP7, with minimal off-target liabilities, high oral bioavailability, and a NOAEL of 1,000 mg/kg/day in rats and dogs. In an established preclinical OIPN model, acute oral dosing (25–100 mg/kg) showed a cannabinoid receptor type 1 (CB_1_)-dependent anti-allodynic effect lasting up to 8 hours (persisting longer than plasma exposure to ART26.12). Antagonists of cannabinoid receptor type 2 (CB_2_), peroxisome proliferator-activated receptor alpha, and transient receptor potential cation channel subfamily V member 1 (TRPV1) may have also been implicated. Twice daily oral dosing (25 mg/kg bis in die (BID) for 7 days) showed anti-allodynic effects in an established OIPN model without the development of tolerance. In a prevention paradigm, coadministration of ART26.12 (10 and 25 mg/kg BID for 15 days) with oxaliplatin prevented thermal hyperalgesia, mitigated mechanical allodynia, and attenuated OXA-induced weight loss. Multi-scale analyses revealed widespread lipid modulation, particularly among *N*-acyl amino acids in the spinal cord, including potential analgesic mediators. Additionally, ART26.12 administration led to upregulation of ion channels in the periaqueductal grey, and broad translational upregulation within the plasma proteome. These results show promise that ART26.12 is a safe and well-tolerated candidate for the treatment and prevention of OIPN through lipid modulation.

**Perspective:**

Inhibition of fatty acid-binding protein 5 (FABP5) is a novel target for reducing pain associated with chemotherapy. ART26.12 is a safe and well-tolerated small molecule FABP5 inhibitor effective at preventing and reducing pain induced with oxaliplatin through lipid modulation and activation of cannabinoid receptors.

Chemotherapy-induced peripheral neuropathy (CIPN) is a common side effect of multiple cytotoxic chemotherapeutic agents (eg, platinums, taxanes, vinca alkaloids etc.). CIPN is characterised by mechanical allodynia and thermal hyperalgesia in the extremities. Close to 70% of all cancer patients will develop CIPN within the first month.^1^ This may severely limit or terminate treatment, leading to chronic debilitating morbidity. This dose-limiting toxicity has implications for both patient outcomes and healthcare costs.^2^ Given that a higher percentage of patients are now surviving cancer,^3^ it is likely that the incidence of CIPN will increase.

Oxaliplatin (OXA) is a platinum-based anti-neoplastic agent that causes CIPN (specifically known as Oxaliplatin-induced peripheral neuropathy [OIPN]). Acute OIPN occurs in up to 98% of patients treated with OXA.^4,5^ Common symptoms of OIPN include sensitivity to cold, paraesthesia in the throat, difficulty swallowing, and muscle fasciculations/contractions.^6^ Patients suffering from chronic OIPN may also present with impaired sensorimotor function. It is estimated that between 20 and 50% of patients receiving OXA treatment will develop chronic OIPN.^6–8^ “Coasting” is a phenomenon where the symptoms of OIPN worsen in the months following treatment cessation.

The pathophysiology of OIPN is complex. OXA and other platinum-based agents are thought to accumulate in the dorsal root ganglion (a collection of sensory neurons which transmit signals from the periphery to the central nervous system), which are not protected by the blood-brain barrier.^9^ Acute OIPN is mediated through the dysfunction of voltage-gated ion channels (eg, sodium, potassium, and calcium), transient receptor potential channels, organic cation transporters 1 and 2, Mate 1 transporter, and glial cells.^10,11^ Chronic OIPN is attributed to DNA and mitochondrial damage, reactive oxygen species causing oxidative stress, and neuro-immune activation causing neuroinflammation.^10^ Currently, there are no licensed medications for the treatment or prevention of OIPN.

FABPs are intracellular chaperones of lipophilic molecules, including endocannabinoids (eCBs) and other *N*-acylethanolamines (NAEs).^12–14^ Whilst there are 10 human FABP isoforms, FABP3, FABP5, and FABP7 are predominantly expressed throughout the central and peripheral nervous systems.^15^ CB_1_, eCB enzymes linked to synthesis and degradation, and TRPV1 are expressed in peripheral nociceptors.^16–22^ Anandamide (AEA) is an eCB that exerts anti-nociceptive and anti-inflammatory effects via activation of CB_1_, CB_2_, and/or TRPV1 in several different pain models.^23–25^ Other NAEs, including oleoylethanolamide and palmitoylethanolamide (PEA), exert anti-nociceptive and anti-inflammatory effects through activation of peroxisome proliferator-activated receptor alpha (PPARα).^25–27^ Numerous studies have shown that inhibition or deletion of FABP5 has anti-nociceptive effects in inflammatory pain models.^25,27–32^ This is thought to be mediated by reduced transport of AEA, oleoylethanolamide, and PEA to their catabolic enzymes, thus reducing and increasing their metabolism and tone, respectively.

Given the localisation of FABP5 in the DRG,^27,32^ its role in the transportation of NAEs, the anti-nociceptive and anti-inflammatory effects of these NAEs, and evidence that exogenous synthetic and phytocannabinoids can ameliorate the effects of OIPN, the investigation of FABP5 inhibition in preclinical rat models of OIPN is warranted. This study aimed to assess whether a novel FABP5 inhibitor (ART26.12) has acute effects on established OIPN, can treat established OIPN, and whether ART26.12 can prevent the onset of OIPN.

## Methods

### FABP Binding

The in vitro binding affinity inhibition constant (K_i_) of ART26.12 to FABP3, FABP4, FABP5, and FABP7 was evaluated in a fluorescence displacement assay. Purified FABPs were incubated with 11-(dansylamino)undecanoic acid (DAUDA) (FABP3, FABP5) or 8-anilino-1-naphthalenesulfonic acid (ANS) (FABP7) in the presence or absence of FABP inhibitors (.01–200 μM) in the binding assay buffer. FABP4 binding was assessed using a commercially available kit (Cayman Chemical, Ann Arbor). In all cases, arachidonic acid (10 μM) was included to account for maximal probe displacement. Loss of fluorescence intensity was monitored with an F5 Filtermax Multi-Mode Microplate Reader (Molecular Devices, Sunnyvale) using the following excitation and emission wavelengths (DAUDA: ex./em.= 360/535 nm; ANS: ex./em. = 360/465 nm). Following background subtraction, the fluorescence intensity values were normalised and analyzed via a one-site binding model using the GraphPadPrism (version 9.2). Ki values were determined using the following equation: Ki= IC50/(1 + ([Probe]/Kd).

### Oral Bioavailability

The oral bioavailability of ART26.12 in male Sprague Dawley rats was determined by acute dosing of ART26.12 by oral (PO, 10, or 300 mg/kg) and intravenous (IV, 2 mg/kg) administration. Following intravenous and oral administration, blood samples (ca .2 mL) were collected from the jugular vein. For IV group, blood samples were collected at .083, .25, .5, 1, 2, 4, 6, 8, 24 hours post-dose. For PO group, blood samples were collected at .25, .5, 1, 2, 4, 6, 8, 24 hours post-dose. Blood samples were collected into tubes containing the anticoagulant K_2_EDTA. Plasma samples were prepared by centrifugation and dispensed into 1.5 mL matrix tubes and stored in a freezer (−75 ± 1°C) prior to analysis.

### Safety and Toxicity

ART26.12 was evaluated using in vitro binding and enzymatic and uptake panels totalling 87 pharmacological targets (receptors, ion channels, and enzymes) at a test concentration of 30 µM. Findings were considered significant if ≥50% inhibition of ligand binding or ≥50% stimulation/inhibition were shown in biochemical assays. Those receptors with values ≥50% were further evaluated for binding and functional half-maximal inhibitory concentrations (IC_50_).

ART26.12 was evaluated in a 14-day repeated dose toxicity study in Wistar Han rats to establish a no observed adverse effect level (NOAEL) up to 1,000 mg/kg/day. Previous results indicated that 1,000 mg/kg/day was tolerated as a single dose. Twenty-nine male and 29 female Wistar Han rats were assigned to 7 groups (4 main and 3 toxicokinetic (TK) groups), with 5 rats/sex/group in the main groups and 3 rat/sex/group in TK groups. Dose formulations were administered twice daily by oral gavage at 100, 300, and 1,000 mg/kg/day (50, 150, and 500 mg/kg/dose) for 14 consecutive days, with a vehicle control group dosed in the same manner. Toxicity was assessed based on clinical signs, daily body weights, food consumption, organ weights, clinical, gross, and anatomic pathology.

Following this, ART26.12 was evaluated in a 14-day repeated dose toxicity in beagle dogs to establish a NOAEL in a second species. Naïve dogs (1/sex/group) were given a PO dose of vehicle control or ART26.12 at 100 mg/kg/day (50 mg/kg/dose), 300 mg/kg/day (150 mg/kg/dose), and 1,000 mg/kg/day (500 mg/kg/dose) for 14 consecutive days. Examined parameters to assess toxicity included clinical observations, mortality and moribundity cheques, body weights, food consumption, electrocardiogram, TK, clinical chemistry, haematology, coagulation, urinalysis, gross pathology, organ weights, and histopathology.

### Potential Analgesic Effects of ART26.12 in OIPN

OXA (Tocris Bioscience) was dissolved in 5% dextrose (Sigma-Aldrich) and administered intraperitoneally (IP) at a volume of 6 mL/kg. ART26.12 (Charnwood Molecular) was dissolved in 5% dimethyl sulfoxide and 20% Vitamin E D-α-Tocopherol polyethylene glycol 1,000 succinates in deionised water and was administered PO. Pregabalin (O’Chem Inc) was dissolved in deionised water and administered PO. AM251 (CB_1_ antagonist; Tocris Bioscience), AM630 (CB_2_ antagonist; Tocris Bioscience), GW6471 (PPARα antagonist; Tocris Bioscience), and AMG9810 (TRPV1 antagonist; Tocris Bioscience) were dissolved in saline with 4% dimethyl sulfoxide and delivered IP.

Male Sprague Dawley rats (150–210 g) were housed in groups of 4 in intra-ventilated Perspex home cages. Humidity, temperature (21 ± 1 °C), and lighting (light-dark cycle of 12:12 hours) were controlled and maintained in the home cages, with food and water available ad libitum. Rats acclimatised to the home cages for at least 1 week. All experiments were carried out according to the Animals (Scientific Procedures) Act, 1986 (U.K. Government Home Office). The study was performed under the Home Office project licence number PP0847012. OIPN was established with a single dose of OXA (10 mg/kg, IP).

In already established OIPN, a dose-response to ART26.12 (25, 50, 75, and 100 mg/kg, PO) was investigated with pain measurements at 30 minutes and 2 hours post-dose. The potential receptor mechanisms of action were assessed by co-administering antagonists to CB_1_ (AM251 3 mg/kg, IP), CB_2_ (AM630 3 mg/kg, IP), PPARα (GW6471 4 mg/kg, IP) and TRPV1 (AMG9810 3 mg/kg, IP) with ART26.12 (100 mg/kg, PO); pain measurements were taken at 30 minutes and 2 hours post-dose. The doses were based on previous studies that used these antagonists in animal models of CIPN.^33–37^ These doses were shown to not affect CIPN when administered alone.^33,37^ The time course of the analgesic response to a single dose of ART26.12 (100 mg/kg, PO) was assessed in another study, with serial tail vein bleeds at the same time points as pain behaviour measurements (.5, 1, 2, 3, 4, 8, and 24 hours post-dose).

To test whether repeated dosing reduces pain in OIPN, animals with already established neuropathy were treated for 7 days with ART26.12 (10 mg/kg, PO, QD, 25 mg/kg, PO, QD, 50 mg/kg, PO, QD or 25 mg/kg, PO, bis in die (BID)) or the positive control pregabalin (30 mg/kg, PO, Day 7 and 14). Pain measurements were made 2 hours after the first dose of ART26.12 and 2 hours after the final dose on day 14. Body weight was monitored throughout the study, and terminal plasma and whole brain samples were taken for bioanalysis.

The final study examined the ability of ART26.12 to prevent the induction of neuropathy. ART26.12 (10 or 25 mg/kg, PO, BID) and pregabalin (30 mg/kg, PO, QD) treatment began on the day of OXA administration for 14 days. Pain measurements were made on Days 5/6 (when neuropathy has normally developed) and at the end of the study. Pain measurements were made 2 hours after ART26.12 dosing. Terminal plasma samples were taken for bioanalysis and proteomics (Somalogic). Brain regions involved in pain processing, that is, prefrontal cortex (PFC), periaqueductal grey (PAG), whole spinal cords (SC), and L4-L6 dorsal root ganglia, were collected for lipidomic and genomic (Genewiz, China) analyses.

Tactile allodynia (primary outcome measure) was assessed using paw withdrawal threshold (PWT), a measure of pain sensitivity. PWT was evaluated using calibrated von-Frey monofilaments (Touch-Test Sensory Evaluator; Scientific Marketing Associates) applied to the plantar surface of the left hind paw. PWT was determined by increasing and decreasing stimulus intensity and estimated using Dixon’s up-down method.^38,39^

Thermal hyperalgesia was assessed using a cold plate (Bioseb, France) set at a fixed temperature of 15 °C. Latency to the first escape behaviour from the plate was measured indicating discomfort to the thermal stimulation. Escape behaviours typically consist of paw licking, jumping, or withdrawal. The maximal threshold of latency was set at 150 seconds.

To acquire baseline readings, pain behaviour was assessed on 3 consecutive days (Days −3, −2, −1). The mean of Day −2 and Day −1 was considered the healthy baseline (HBL) prior to OIPN induction. On Days 5 and 6 after OXA injection, thresholds were reassessed. The mean of Days 5 and 6 was considered the neuropathic baseline (NPB). Animals were then ranked and randomised (based on a Latin square design) to treatment groups according to the percentage change of the mean thresholds observed in the NPB compared to the HBL. NPB expressed as a percentage change from HBL was calculated as follows (HBL-NPB)/HBL*100. All behavioural assessment was done by an experimenter blinded to the identity of the treatment. Separate people were responsible for dosing and assessment/data analysis.

### Plasma Levels of ART26.12

Quantitative bioanalysis by LC-MS/MS of plasma and spinal cord levels of ART26.12 was carried out by a commercial supplier (Sygnature Discovery, Nottingham, UK).

### Lipidomics

Lipid extraction from tissues was performed as previously described.^40^ Samples were flash frozen in liquid nitrogen, weighed and transferred to a centrifuge tube with HPLC-grade methanol (Thermo Fisher Scientific, Fair Lawn, NJ). Tubes were spiked with 500 picomoles d_8_AEA (Cayman Chemical, Ann Arbor, MI). Samples were left on ice for 2 hours, homogenised and centrifuged at 19,000 g for 20 minutes at 20°C. Supernatants were retained and diluted with 3 volumes of HPLC water (Fisher). Lipids were partially purified on C-18 solid phase extraction columns (Agilent, Palo Alto, CA). Elution samples were then analysed by HPLC MS/MS as previously described.^40^ 20 μL samples were run on an Agilent XDB-C18 reversed-phase analytical column and analysed using an Applied Biosystems API 3,000 triple quadrupole mass spectrometer with electrospray ionisation (Foster City, CA). The lipid library was interrogated using multiple reaction monitoring as previously described.^40^ Concentrations in mols/gram were adjusted according to extraction efficiency, as determined by d_8_AEA recovery. For each individual lipid species, samples were unblinded, fold-change compared to the vehicle was calculated and significance calculated by t-test (*P* < .05 and *P* < .1) in R.

### Genomics

Sequencing (Illumnia NovoSeq) was done at Genewiz (Shanghai, China). Sequence reads were trimmed using Trimmomatic (v.0.36). Reads were then aligned to the *R. norvegicus* Rnor6.0 reference genome (https://www.ncbi.nlm.nih.gov/assembly/GCF_000001895.5/) using the STAR aligner (v.2.5.2b) and unique gene hit counts were calculated using featureCounts from the Subread package (v.1.5.2). Differentially expressed genes were determined using limma 3.46.0. Genes were considered significant if they had a fold change ≥1.5 and false-discovery rate (by Benjamini-Hochberg method) adjusted *P*-value ≤.05. Visualisation was done using the EnhancedVolcano (1.7.10) package in R. Gene set enrichment analysis was carried out using the gprofiler2 package in R.

### Proteomics

Plasma protein levels were measured by Somalogic using the SomaScan proteomic assay, an aptamer-based method. SOMAmers are Slow Off-Rate Modified Aptamers, able to capture proteins in a microarray format. Briefly, plasma samples were transferred to Agilent slides, hybridised with SOMAmer reagents, which were subsequently washed and scanned. Over 7,000 plasma proteins were characterised. Samples were normalised using hybridisation controls. Following this, median signal normalisation was carried out across pooled calibrator replicates within runs. Plate scaling was done to adjust for overall signal intensity differences between runs. Samples were then calibrated to adjust for SOMAmer reagent-specific assay differences between runs. Median signal normalisation was performed using Adaptive Normalisation by Maximum Likelihood. No samples were flagged as outliers by quality control.

Samples were unblinded and data analysis was done within R. Volcano plots are plotted as log2fold change vs log10 *P*-value, calculated by t-test. Visualisation was done using the EnhancedVolcano (1.7.10) package in R. Gene set enrichment analysis was carried out using the gprofiler2 package in R.

### Data Analysis

Data were analysed in Prism (version 9 for Mac, GraphPad Software, La Jolla, CA). Values that were outside the range of mean ± 3 standard deviations were considered outliers and excluded from the analysis. Where 2 time points were compared, a paired, two-tailed t-test was applied. Where 3 or more time points were compared, a one-way repeated measures analysis of variance was applied followed by a Tukey’s post-hoc test to determine differences between time points. When comparing between 3 or more treatment groups, a two-way repeated measures analysis of variance was applied. Tukey’s post-hoc test followed to determine differences between all treatment groups, whilst Dunnett’s or Šidák’s post-hoc tests were applied to determine differences between treatment groups and vehicles. Values are presented as mean ± standard error of the mean.

## Results

### ART26.12 Drug Characteristics

**Figure 1.**
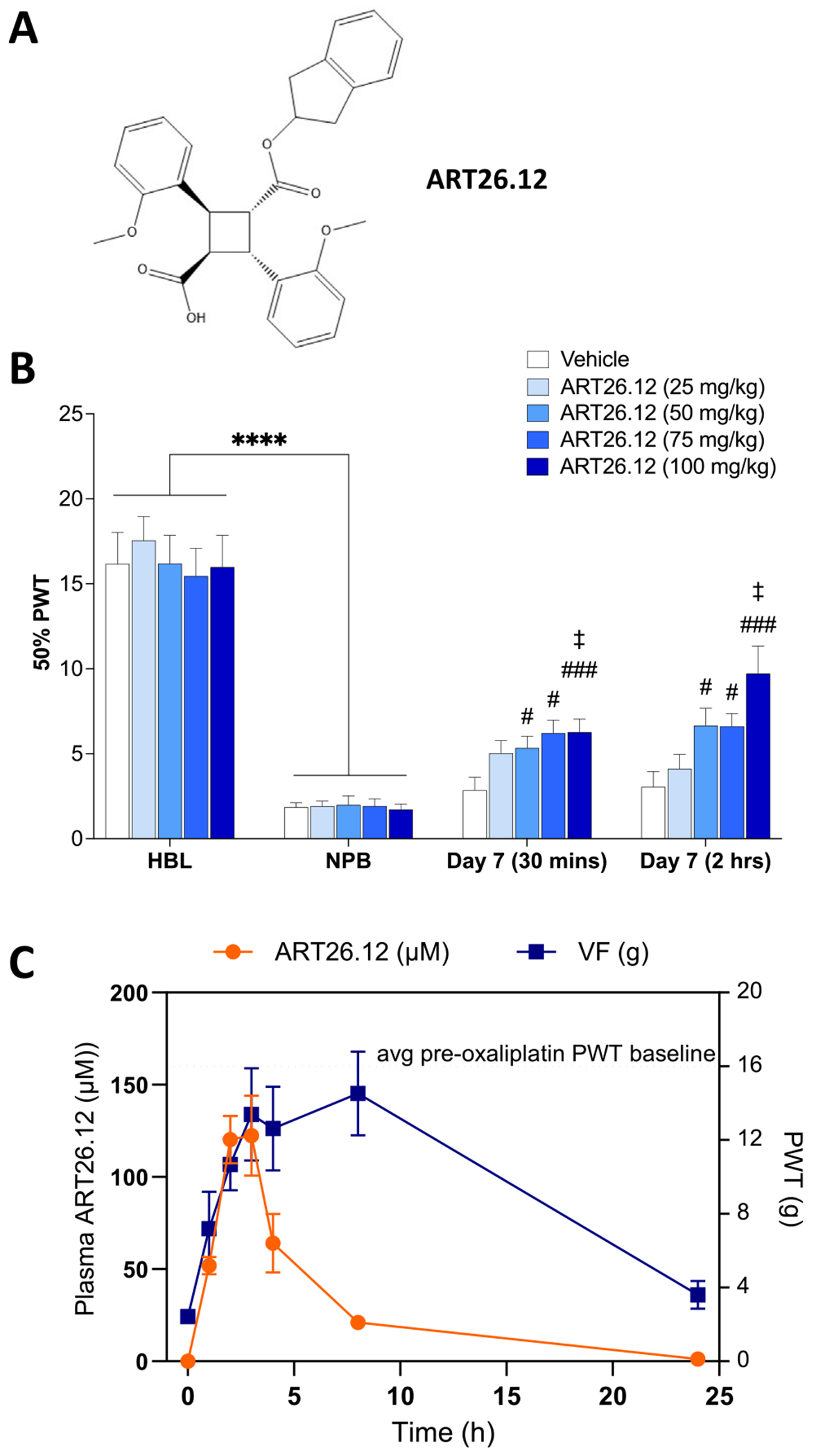
The dose- and time-dependent analgesic effects of ART26.12 on OIPN. Structure of ART26.12, an FABP5 selective inhibitor **(A)**. OIPN was induced by a single IP dose of OXA on Day 0 with HBL and NPB measured on Days −1/−2 and Days 5/6, respectively. ART26.12 was acutely administered PO on Day 7 at multiple doses with PWT measured at 30 min and 2 h post-dose; n = 10 **(B)**. PWT was analysed over time with a matching pharmacokinetic analysis of ART26.12 plasma concentration; n = 12 (C). ****P < .0001 when comparing between a healthy baseline and a neuropathic baseline. #*P* < .05, ###*P* < .001 when comparing between each drug treatment and vehicle. ‡*P* < .05 when comparing between ART26.12 (100 mg/kg) and ART26.12 (25 mg/kg). HBL, healthy baseline; IP, intraperitoneally; NPB, neuropathic baseline; OIPN, oxaliplatin-induced peripheral neuropathy; OXA, oxaliplatin; PO, orally; PWT, paw withdrawal threshold; VF, von-Frey.

ART26.12 ((+)–3-Indan-2-yloxycarbonyl-2,4-bis(2-methoxyphenyl)cyclobutanecarboxylic acid) is a third-generation FABP5 selective inhibitor from the truxillic acid monoester series of FABP inhibitors (Fig 1A). It shows comparable potency to the first-generation compound SBFI-26, with improved selectivity. The α-truxillic acid scaffold has been modified to include 2-methoxy-phenyl derivatives on the cyclobutane ring. It displays favourable druglike properties satisfying Lipinksi’s Rule of 5 (cLogP: 4.5, Molecular weight: 472.5 g/mol, H-bond donors: 1, H-bond acceptors: 6).^41^ ART26.12 showed selectivity for FABP5 (Ki .77 ± .08 μM) over FABP3 (71.32 ± 2.40 μM), FABP4 (> 100 μM), or FABP7 (18.99 ± 1.81 μM).

Single-dose oral administration of ART26.12 showed high bioavailability in male rats (73% at 10 mg/kg). At 10 mg/kg, T_max_ was ~.5 hour, indicating rapid absorption. Intravenous administration (2 mg/kg) showed low plasma clearance (14 mL/minute/kg), modest volume of distribution (1.3 L/kg), and a short half-life (2.6 hours). At 300 mg/kg, T_max_ was ~6.6 hours, half-life was 3.8 hrs and bioavailability was 163%.

In secondary pharmacology screening, ART26.12 was found to bind to 4 targets with values greater than 50% (CCK_1_ [cholecystokinin A receptor], CCK_2_ [cholecystokinin B receptor], δ [DOP; delta opioid receptor], and cathepsin G). Subsequent functional assessment at these receptors suggests these interactions were not physiologically relevant.

No mortalities, clinical signs of systemic toxicity, or histopathological effects were observed throughout 14-day consecutive oral dosing of ART26.12 in male and female Wister Han rats at the doses of 100, 300, and 1,000 mg/kg/day (50, 150, and 500 mg/kg BID). No body weight changes were observed at 100 or 300 mg/kg/day, with a slight decrease on days 1 to 4 at 1,000 mg/kg/day in both sexes. Similarly, 14-day consecutive PO dosing of ART26.12 in male and female beagle dogs showed no mortalities and no signs of toxicity up to and including 1,000 mg/kg/day (500 mg/kg BID). Hence, a NOAEL of 1,000 mg/kg/day (500 mg/kg BID) was established in both species.

### Potential Analgesic Effects of ART26.12 in OIPN

#### Dose-Dependent Effects of Acutely Dosed ART26.12 on Oxaliplatin-Induced Mechanical Allodynia

In established OIPN, ART26.12 significantly increased PWT when compared to vehicle 30 minutes and 2 hours post-dose (at 50 mg/kg (*P* < .05), 75 mg/kg (*P* < .05), and 100 mg/kg (*P* < .001), Fig 1B).

#### Pharmacokinetic and Pharmacodynamic (PKPD) Profile of ART26.12’s Anti-allodynic Effect

In established OIPN, after a single dose of ART26.12 (100 mg/kg), PWT was significantly increased at 30 minutes (*P* < .05), 2 hours (*P* < .01), 3 hours (*P* < .05), 4 hours (*P* < .05), and 8 hours (*P* < .01, Fig 1C). Bioanalysis of plasma samples data from the same animal cohort shows that the total plasma concentration of ART26.12 increases steadily to a peak at around 3 hours (122 ± 13 µM, mean ± SEM), steadily reducing by 8 hours, and back to baseline by 24 hours (Fig 1C).

#### ART26.12’s Anti-allodynic Mechanism of Action

**Figure 2.**
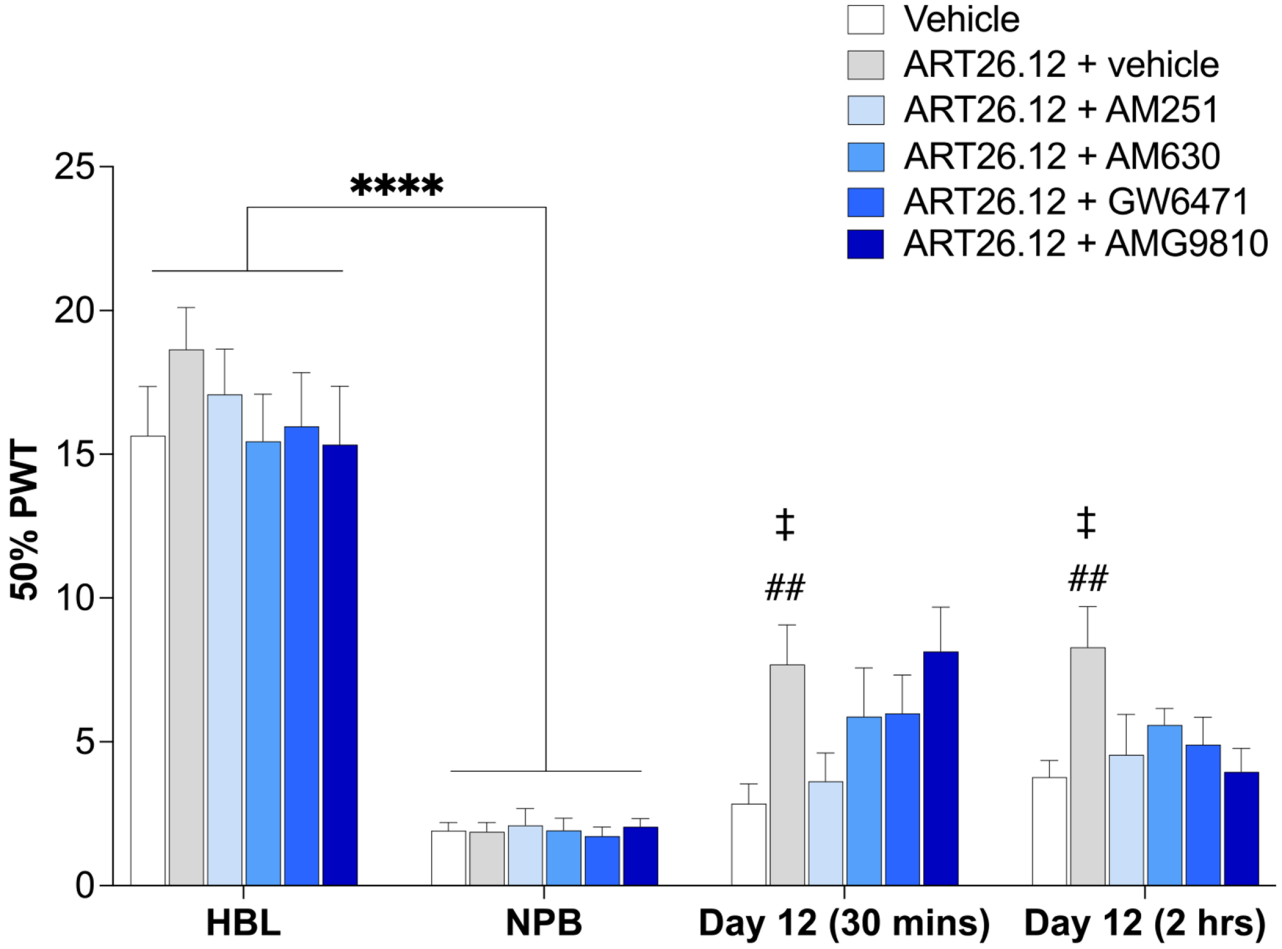
The receptor mechanism of action underpinning acute ART26.12 analgesia in OIPN. OIPN was induced by a single IP dose of OXA on Day 0 with HBL and NPB measured on Days −1/−2 and Days 5/6, respectively. ART26.12 (100 mg/kg PO) was administered with and without various receptor antagonists (AM251 (CB_1_ antagonist), AM630 (CB_2_ antagonist), GW6471 (PPAR-alpha antagonist), and AMG9810 (TRPV1 antagonist), all administered 10 min before ART26.12. Vehicle, ART26.12 + AM251, and ART26.12 + AMG9810 n = 11, all other groups n = 12. ****P < .0001 when comparing between healthy baseline and a neuropathic baseline. ##*P* < .01 when comparing between vehicle and ART26.12 + vehicle. ‡*P* < .05 when comparing between ART26.12 + vehicle and ART26.12 + AM251. HBL, healthy baseline; IP, intraperitoneally; NPB, neuropathic baseline; OIPN, oxaliplatin-induced peripheral neuropathy; OXA, oxaliplatin; PO, orally; PWT, paw withdrawal threshold.

In established OIPN, an acute dose of ART26.12 (100 mg/kg) significantly increased PWT when compared to vehicle-alone (*P* < .01) at 30 minutes or 2 hours, as previously observed. Animals co-treated with ART26.12 and AM251 had significantly reduced PWT when compared to ART26.12 alone (*P* < .05, Fig 2). When ART26.12 was co-administered with either AM630, GW6471, or AMG9810, the ability of ART26.12 to increase PWT was reduced such that these responses were not significantly different to vehicle at the 2 hours time point (Fig 2).

#### Chronic Treatment of Oxaliplatin-Induced Pain Behaviours with ART26.12

**Figure 3.**
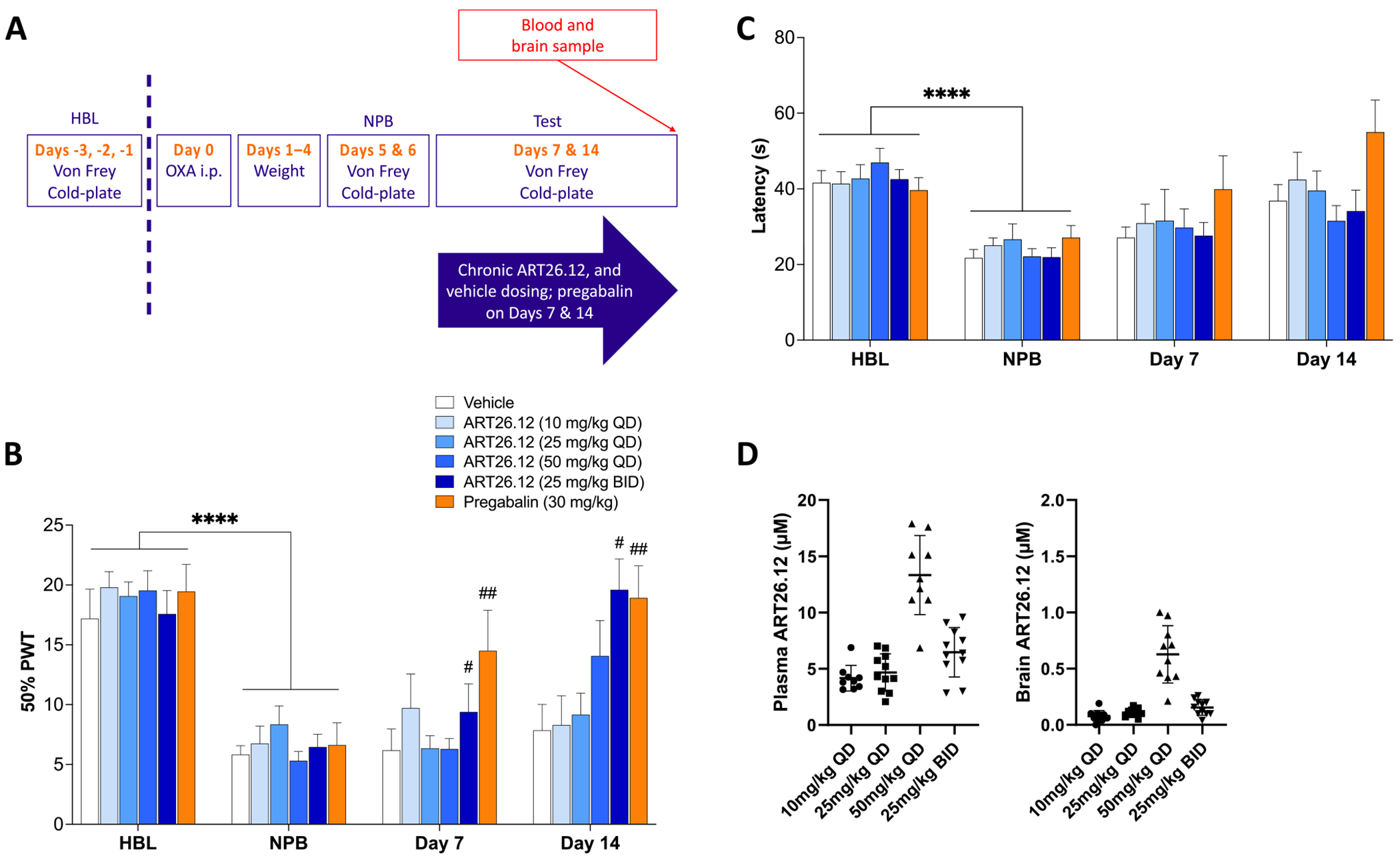
Treatment of established OIPN with various doses of ART26.12. OIPN was induced by a single IP dose of OXA on Day 0 with HBL and NPB measured on Day −1/−2 and Days 5/6, respectively. ART26.12 was chronically administered PO from Days 7 to 14 at multiple doses, whilst pregabalin was dosed acutely only on Days 7 and 14 **(A)**. PWT and cold plate latencies were measured on Days 7 and 14 **(B, C)**. Plasma and brain concentrations of ART26.12 samples were taken on Day 14 **(D)**. Pregabalin n = 8, ART26.12 (25 mg/kg QD) n = 11, all other groups n = 10. ****P < .0001 when comparing between healthy baseline and a neuropathic baseline. #*P* < .05, ##*P* < .01 when comparing between drug treatment and vehicle. HBL, healthy baseline; IP, intraperitoneally; NPB, neuropathic baseline; OIPN, oxaliplatin-induced peripheral neuropathy; OXA, oxaliplatin; PO, orally; PWT, paw withdrawal threshold.

A schematic of the protocol of this study is shown in Fig 3A. In established OIPN, across Days 7 and 14, ART26.12 administered at 25 mg/kg BID (*P* < .05) and pregabalin (*P* < .01) showed significantly higher PWT scores than vehicle (Fig 3B). ART26.12 had no effect on cold plate latency, however, it is important to note that by Day 14, the vehicle group showed cold plate latencies that were close to their HBL levels (Fig 3C). Terminal plasma samples showed an average total plasma concentration of 6.5 ± .6 µM after 7 days dosing with ART26.12 25 mg/kg BID, and a total brain concentration of .15 ± .02 µM (Fig 3D).

#### Prevention of Oxaliplatin-Induced Pain Behaviours with Chronically Dosed ART26.12

**Figure 4.**
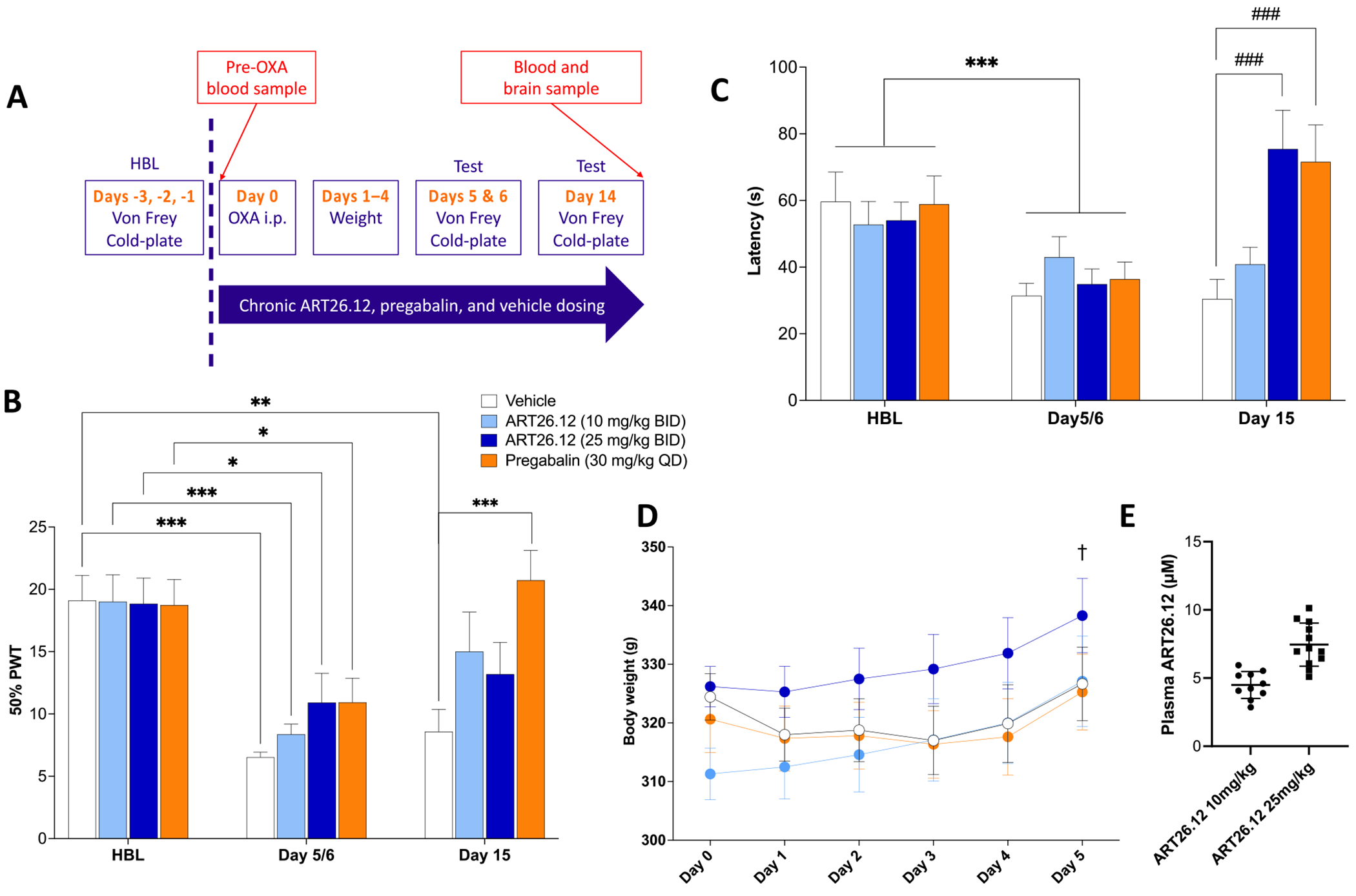
Prevention of OIPN with chronic dosing of ART26.12. OIPN was induced by a single IP dose of OXA on Day 0 with HBL measured on Days −1/−2, respectively. ART26.12 and pregabalin were chronically administered PO from Days 0 to 14 at multiple doses **(A)**. PWT and cold-plate latencies were measured on Days 5/6 and 14 **(B, C)**. Bodyweight measurements taken on Days 1 to 14 **(D)**. Plasma and brain concentrations of ART26.12 were measured on Day 14 **(E)**. Vehicle n = 9, ART26.12 (10 mg/kg BID) n = 10, ART26.12 (10 mg/kg BID) n = 12, pregabalin n = 11. *P < .05, **P < .01, ***P < .001 when comparing between healthy baseline and Day 7. ###*P* < .001 when comparing between drug treatment and the respective day’s vehicle. HBL, healthy baseline; IP, intraperitoneally; OIPN, oxaliplatin-induced peripheral neuropathy; OXA, oxaliplatin; PO, orally; PWT, paw withdrawal threshold.

A schematic of the protocol of this study is shown in Fig 4A. On Day 5/6, the vehicle (*P* < .001), ART26.12 10 mg/kg BID (*P* < .001), 25 mg/kg BID (*P* < .05), and pregabalin (*P* < .05) groups showed significantly lower PWTs when compared to HBL. However, only the vehicle group showed a significantly lower PWT when comparing between Day 14 and HBL, whilst neither the ART26.12 nor the pregabalin groups showed significant allodynia at this point (Fig 4B). On Day 14, animals treated with ART26.12 (25 mg/kg BID; *P* < .001) or pregabalin (*P* < .001) showed significantly higher cold plate latencies than the vehicle group (Fig 4C).

Chemotherapies commonly induce weight loss, which was observed in the vehicle and pregabalin groups only in the first 4 days post-administration of OXA. On Day 5, the ART26.12 (10 mg/kg BID) group showed a significantly higher body weight than Day 0 (*P* < .05), which was not seen in the other groups (Fig 4D). This may have been due to a lower average body weight of the 10 mg/kg group at the start of the experiment.

Mean plasma levels for ART26.12 on Day 15 of the study were 4.49 ± .99 μM in the ART26.12 (10 mg/kg BID) group and 7.45 ± 1.58 μM in the ART26.12 (25 mg/kg BID) group (Fig 4E). Terminal tissues from Day 15 were taken for transcriptomic, lipidomic, and proteomic analyses.

#### Transcriptomic Analysis of Dorsal Root Ganglion, Periaqueductal Grey, and Prefrontal Cortex Tissue

**Figure 5.**
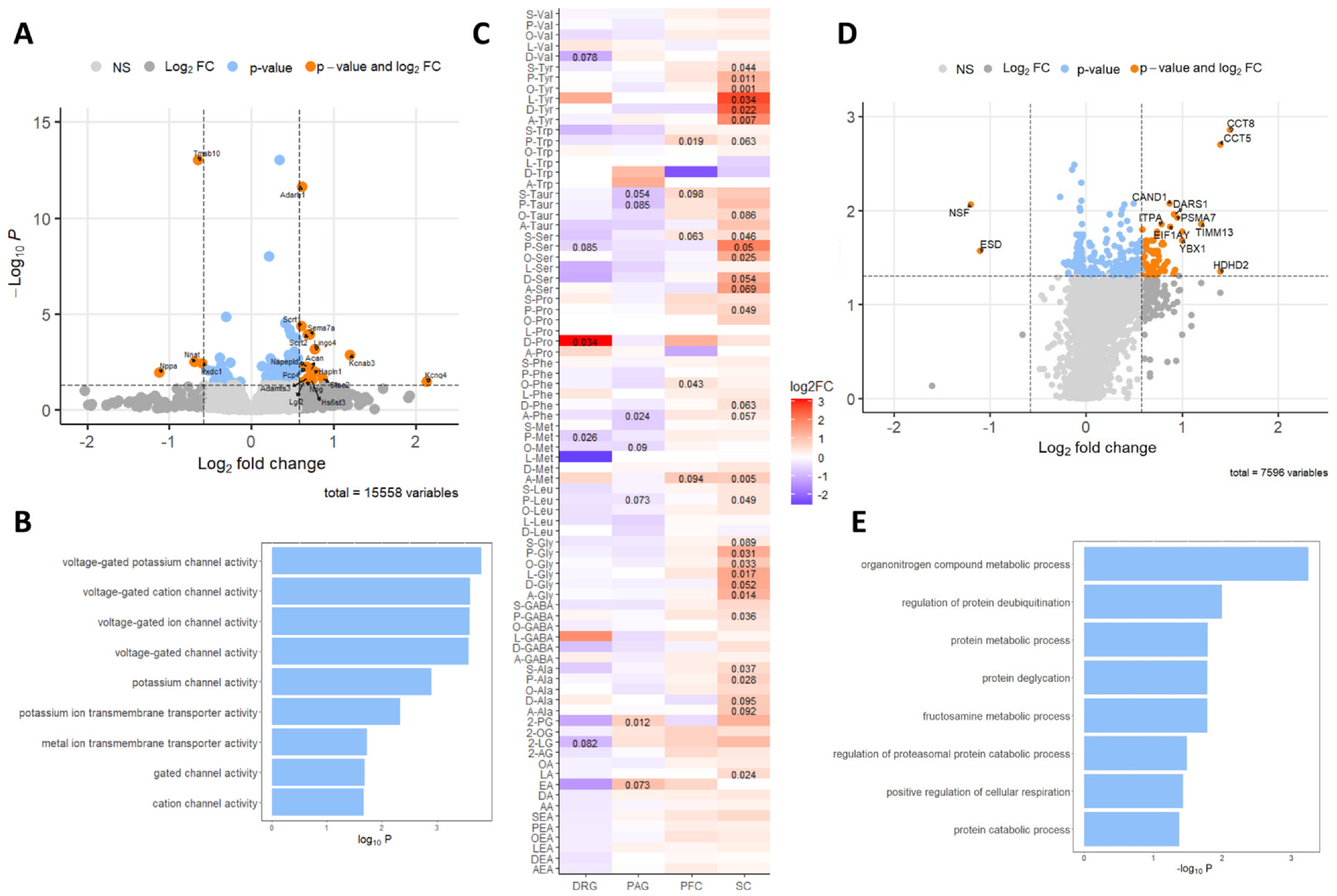
Multi-omic analysis of terminal tissues and plasma following prevention of OIPN with chronic dosing of ART26.12. Volcano plot of RNA-seq analysis of terminal tissue from the periaqueductal grey (PAG), log2FC > .58, *P* < .05 n = 5/group **(A)**. GO enrichment analysis of the molecular function of all significantly changed genes in the PAG (*P* < .05) **(B)**. Heatmap of lipidomic analysis in the dorsal root ganglion (DRG), PAG, prefrontal cortex (PFC), and spinal cord (SC). Values expressed as log2FC over vehicle treatment, significant *P* values shown (*P* < .1). Lipids clustered by family **(C)**. Volcano plot of plasma proteomes of ART26.12 (25 mg/kg) treated rats, compared to vehicle-treated rats. log2FC > .58, *P* < .05, n = 5/group **(D)**. Volcano plot of plasma proteomes of ART26.12 (10 mg/kg) treated rats, compared to vehicle-treated rats. log2FC > .58, *P* < .05 n = 10/group **(E)**. Gene ontology analysis of significantly changed proteins (ART26.12, 25 mg/kg).

Analysis of the transcriptomes revealed numerous bidirectional changes were observed within the PAG, with few changes observed in other tissues. Within the PAG, 20 significantly different genes were identified (*P* < .05, log2FC > .58) (Fig 5A). Gene Ontology terms highlighted changes in voltage-gated ion channels, particularly potassium channels (Fig 5B). All ion channels that were significantly changed in response to ART26.12 were upregulated. Additionally, genes associated with GABAergic inhibitory synapses were upregulated, and several ribosomal-associated proteins were downregulated. Interestingly given its putative key role in OIPN, few transcriptomic changes were observed within the DRG with only glycine decarboxylase being significantly upregulated (*P* < .05, FC > 1.5). No significant changes were observed within the PFC.

#### Lipidomic Analysis of Dorsal Root Ganglion, Spinal Cord, Periaqueductal Grey and Prefrontal Cortex Tissue

Within an OIPN prevention paradigm, ART26.12 (25 mg/kg BID) led to tissue-specific bidirectional changes in measured lipids, with those measured in the spinal cord showing the most robust changes (Fig 5C). All measured lipids within the spinal cord trended towards upregulation, with many of the *N*-acyl amino acids (NA-AAs) reaching significance. Glycine, tyrosine, alanine, and serine derivatives were particularly concordant, with almost all of these reaching significance. Bidirectional changes were observed within the DRG, with 2-acyl glycerols, free fatty acids, and NAEs trending down. In contrast to the spinal cord, few changes were seen in NA-AAs, with the notable exception of N-docosahexaenoyl proline which was increased 9-fold (*P* = .03). Within the PAG, free fatty acids, 2-acyl glycerols, and NAEs trended up, while NA-AAs trended down. The PFC mirrored changes in the spinal cord with most lipids mildly trending up; however, few reached significance.

#### Plasma Proteomics

Plasma proteomes were compared between the vehicle-treated group at Day 15, the vehicle-treated animals pre-OXA administration, the ART26.12 (10 mg/kg BID) group, and the ART26.12 (25 mg/kg BID) treated group. At both doses (25 mg/kg BID Fig 5D, 10 mg/kg BID data not shown), highly asymmetrical volcano plots were observed, indicating global increases in protein synthesis compared to vehicle. Several factors involved in translation, protein folding chaperones, and elements of the ubiquitin-proteosome system were upregulated at both doses. Gene Ontology analysis was carried out on proteins significantly changed by ART26.12 (25 mg/kg BID; *P* < .05, fold change > 1.5). Among other terms, it was found that the NRF2 signalling pathway was enriched (*P* = .029), as well as elements of the GABAergic synapse (*P* = .048) and proteins with E2F response elements (*P* < .001) (Fig 5E).

During analysis, it was recognised that several proteins were affected by both OXA treatment (comparing Day −1 plasma pre-OIPN induction with Day 15 vehicle treatment, data not shown) and by ART26.12 (comparing 25 mg/kg BID with Day 15 vehicle treatment, Fig 5D). The majority of these were proteins upregulated by OXA, whose expression was further enhanced by ART26.12 with only one protein, NSF, being upregulated by OXA and reversed by ART26.12 (10 mg/kg BID and 25 mg/kg BID). To identify proteins of interest that were not modulated by OXA, we assessed the subset of proteins that were significantly changed in response to ART26.12 (25 mg/kg BID; *P* < .05, FC > 1.5) but were unaffected by OXA. Thirty-six proteins fulfilled this criterial with roles in protein expression and turnover, protein folding, oxidative stress, and DNA repair (particularly, nucleotide excision repair). Enrichment analysis revealed the presence of 5 proteins implicated in peripheral axonal neuropathy (HP database, *P* = .0095) (Table 1).

## Discussion

OIPN is a prevalent, dose-limiting toxicity that can greatly affect quality of life, treatment compliance, and morbidity. However, there are no licensed medications for the treatment and/or prevention of CIPN, demonstrating a clear unmet need. This study aimed to establish whether FABP5 inhibition is a potential novel target for the treatment and prevention of OIPN. We found that a safe, novel, and selective FABP5 inhibitor with good drug-like properties was effective at treating and preventing OIPN in a rodent model. ART26.12 mediated these effects through lipid modulation, particularly endocannabinoids. ART26.12 also prevents paclitaxel-induced peripheral neuropathy (in both male and female rats with similar efficacy) and treats established painful diabetic neuropathy.^42,43^ These data suggest that ART26.12′s mechanism of action may be similar in other neuropathies.

Previous generations of FABP5 inhibitors were found to be effective in multiple pain models.^25,27–32^ We sought to develop a more potent and selective molecule for clinical development. Based on its favourable properties, ART26.12 was chosen as the lead candidate drug for the potential treatment of painful neuropathies. ART26.12 is a well-tolerated, orally bioavailable, FABP5-selective inhibitor with few off-target interactions. Given its tolerability up to 1,000 mg/kg/day in preliminary rodent and non-rodent toxicity studies, the drug candidate offers a potentially large therapeutic window. ART26.12 also has minimal brain penetrance, with a likely peripheral mechanism of action. Previous work has shown that this class of compounds is not associated with addiction or alcohol dependency, which are important characteristics of novel analgesics.^44,45^

In a model of established OIPN, ART26.12 showed acute attenuation of mechanical allodynia that lasted up to 8 hours, exceeding the drug’s plasma exposure. This effect was predominantly mediated by CB_1_, with potential involvement of CB_2_, TRPV1, and PPARα. Dosing ART26.12 for 7 days also reversed mechanical allodynia in an OIPN-treatment model without signs of tolerance. In the OIPN-treatment model, neither ART26.12 nor pregabalin improved cold-plate latency compared to the vehicle. However, thermal hyperalgesia was not maintained in the vehicle group and had reversed to baseline by Day 14, thus ART26.12′s efficacy may have been obfuscated due to the natural resolution of the neuropathy. ART26.12′s mechanism of action is in accordance with previous studies assessing cannabinoids in CIPN, as well as those investigating the anti-nociceptive effects of FABP5 inhibition. Several cannabinoid agonists (eg, Δ-^9^-tetrahydrocannabinol, ACEA, CP55,940, and WIN55,212–2) treat symptoms of cisplatin and paclitaxel-induced peripheral neuropathy in a CB1-dependent manner.^46–53^ Several studies have shown that systemic administration of AEA or PEA, as well as FAAH or MAGL inhibitors, can treat the thermal and mechanical symptoms of CIPN in a CB_1_, CB_2_, and TRPV1-dependent manner.^33,36,54–58^ In the carrageenan model of inflammatory pain, analgesia following FABP5 knockout was mediated by CB_1_, PPARα, and TRPV1, whilst pharmacological inhibition of FABP5 mediated analgesia via CB_1_, CB_2_, and PPARα.^25,27,28,31^ FABP5 knockout and pharmacological inhibition reduce AEA catabolism and increase levels of other NAEs.^25,28^ It is likely that ART26.12 increased NAE tone, which mediated its analgesic effects via these molecular targets.

In the OIPN prevention study, ART26.12 showed robust prevention of thermal hyperalgesia and attenuation of mechanical allodynia. ART26.12 also prevented the acute weight loss associated with OXA treatment, suggesting the benefit of ART26.12 adjunct to chemotherapy may be broader than pain modulation. ART26.12 also led to tissue-specific bidirectional changes in measured lipids, with those measured in the spinal cord showing the most robust changes. Of particular interest is the AEA metabolite *N*-arachidonoyl glycine, which has previously shown analgesic activity when administered intrathecally in rat models of inflammatory and neuropathic pain.^59,60^ Additionally, several TRP channel modulators including the TRPV1 antagonist N-docosahexaenoyl proline, the TRPV2 agonist N-palmitoyl tyrosine, and the TRPV4 agonists N-arachidonoyl tyrosine and N-palmitoyl tyrosine were elevated.^61^ Given their key role in pain processing, with previous work showing that FABP5 conditional knockout in TRPV1+ nociceptors produced analgesia, any of these could be contributing to the anti-nociceptive effect of ART26.12.^27^ Thus, the mechanism of action of ART26.12 is likely to expand beyond modulation of NAEs.

This is the first time FABP5 inhibition has been shown to modulate NA-AAs. The intracellular chaperone of this class of lipids has not been identified; however, it seems likely that some of these would have a high affinity for FABP5. For example, NA-Gly and NAE share a similar structure, where the hydroxyl of the latter is replaced by a carboxylate in the former. Of note the hydroxyl group in NAE forms key hydrogen bonds with Tyr131 and Arg129 in FABP5, the same residues as the carboxylate in SBFI-26 (the first-generation FABP5 inhibitor). This supports the accommodation of this functional group.^14,62^ The bulkiest N-acyl amino acid observed, NA-Tyr, is easily accommodated in the pocket of FABP5 (data not shown), and known structural analogues (OMDM1, OMDM2) have appreciable affinity to FABP5.^13^ This raises the possibility that FABP5 is an intracellular chaperone of NA-AAs and/or their lipid precursors, modulating their localisation and metabolism. Knockout of PM20D1, the major NA-AA metabolic enzyme in mice, led to weight gain and an anti-nociceptive phenotype to chemical and inflammatory but not heat-related pain.^63^ Therefore, the modulation of NA-AAs could be another mechanism contributing to the analgesic activity of ART26.12. Given the relatively low brain penetration, it is surprising that the most robust changes were seen in the spinal cord and could reflect either an alternate tissue of metabolism, or an increased penetration of ART26.12 into the spinal cord as compared to the brain. Confirmation of the efficacy of intrathecal administration would shed further light on the site of action. However, internal studies suggest that the penetration of ART26.12 into the spinal cord is at sufficient levels for target engagement. Given that acute analgesia (within 30 minutes of ART26.12 dosing) is predominately CB1 mediated, coupled with the fact that AEA is unstable and generally synthesised on demand, it is likely that increases in AEA are temporally restricted. The longer-lasting analgesic effects of ART26.12 (which were observed up to 8 hours post-dosing) may be related to non-endocannabinoid lipid modulation.

Widespread transcriptomic changes were observed within the PAG, with very few changes in the DRG or PFC. This was surprising given that FABP5 is well expressed in the DRG, suggesting either that analgesic activity occurs up/downstream of the DRG, or that the mechanism is not reflected within the transcriptome. Given that 8 days of dosing did not show appreciable levels of ART26.12 in the brain, the changes in the PAG likely reflect a modulation of incoming pain signals rather than direct engagement of FABP5. This is consistent with previous studies showing conditional knockout of FABP5 within the PAG did not mediate analgesia.^27^ Among significantly changed genes, *NNat* was significantly downregulated by ART26.12, and this gene was previously shown to be upregulated in response to neuropathic pain.^64^ *Phf24* was also upregulated by ART26.12, which has previously been shown to be downregulated in response to peripheral nerve injury in rats.^65^ Beyond this, broad ion channel upregulation, known to be dysregulated in CIPN,^66^ is indicative of the ameliorative action. For example, *Kcnq4* was one of the most upregulated genes. KCNQ channels are downregulated by OXA,^67^ and the KCNQ channel opener retigabine has shown protection in models of cisplatin-induced peripheral neuropathy.^68^

**Figure 6.**
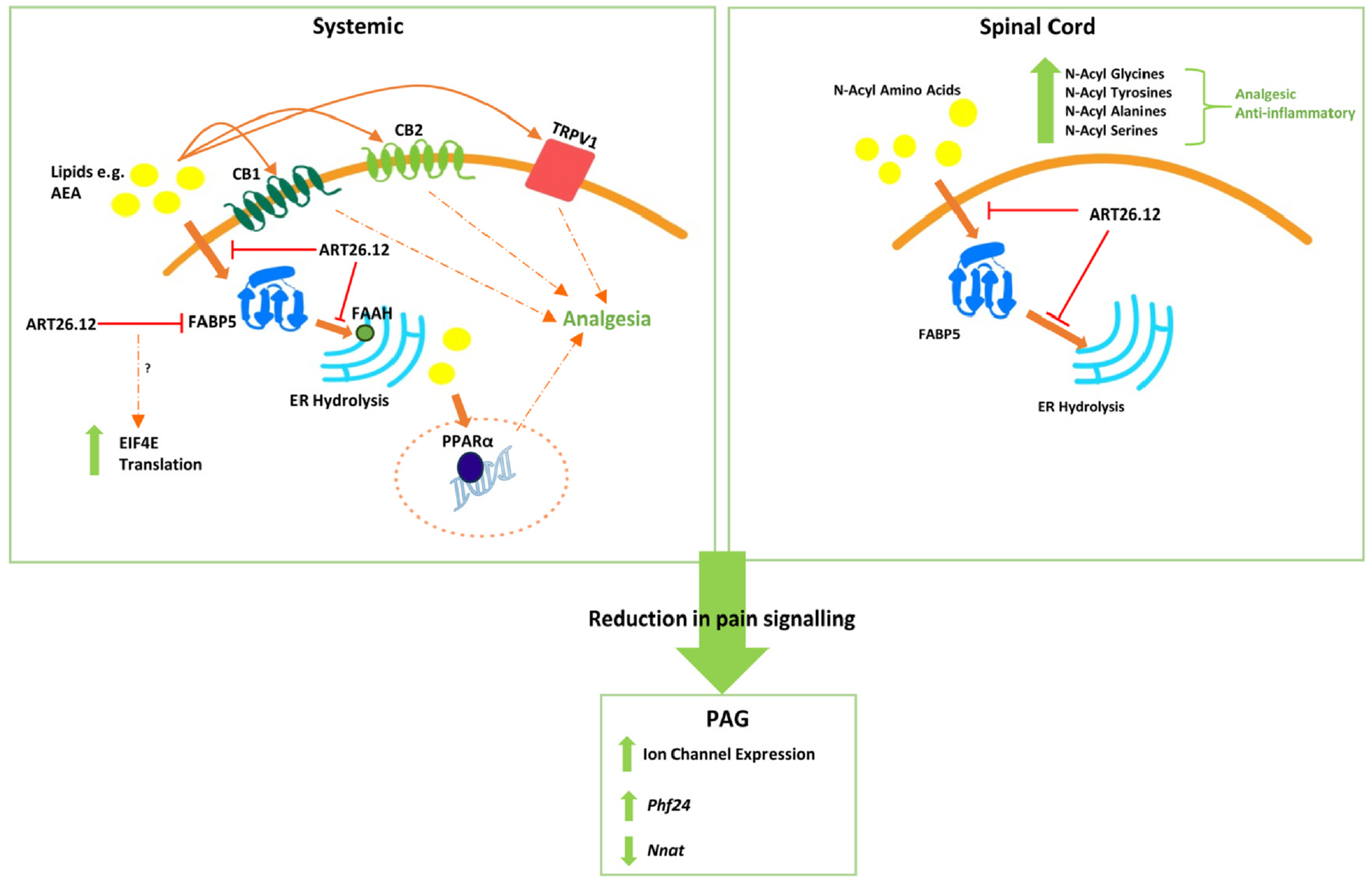
Proposed mechanism of action underlying ART26.12 amelioration of OIPN. Systemically, the acute analgesic effect of ART26.12 was shown to be primarily mediated through CB_1_, with a possible contribution from CB_2_, TRPV1 and PPARα. This is consistent with previous studies which show FABP5 inhibition activates these receptors to produce analgesia by reducing the degradation of AEA and other endocannabinoids, increasing their availability extra- and intra-cellularly (25, 27, 28). Within the spinal cord lipidome, the elevation of numerous analgesic and anti-inflammatory n-acyl amino acids suggested to be by a similar mechanism. Upstream transcriptomic changes within the PAG were observed including increased expression of ion channels, and amelioration of pain marker proteins, suggested to be due to modulation of incoming pain signals.

Within the plasma proteome, we observed an interesting lopsided volcano plot at both doses of ART26.12, suggesting broad protein upregulation. Several factors involved in translation were upregulated at both doses compared to the vehicle. Of particular interest is the rate-limiting translation initiation factor EIF4E, which suggests a possible mechanism for ART26.12 in translational upregulation. OXA treatment downregulates ribosomal biogenesis due to nucleolar dysfunction, presenting a possible explanation for lower translational activity.^69^ It is not obvious how FABP5 inhibition would ameliorate this, and general translational downregulation was not observed when comparing pre-treated plasma with neuropathic plasma. This suggests that the translational upregulation is directly related to FABP5 engagement, rather than upstream amelioration of OXA toxicity. In addition, various protein folding chaperones (including TriC components CCT5 and CCT8 involved in tubulin folding), tRNA synthetases, translation activators, elements of the ubiquitin-proteosome system, and several proteins involved in mRNA transcript stability. These trends were reflected within the Gene Ontology terms, which showed enrichment of proteins involved in RNA binding, ubiquitin ligase activity, and protein binding. A group of 5 proteins were identified that are implicated in peripheral axonal neuropathy. Specifically, point mutations in any of these lead to hereditary degenerative neuropathies highlighting their importance in peripheral neuron survival and maintenance. Interestingly one of these proteins, Valosin-containing protein, was identified as a target for cisplatin adducts along with another significantly upregulated protein OLA1.^70^ However, the significance of their enrichment in the plasma proteome is uncertain, and transcription is not changed within the DRG. The potential mechanisms of analgesia are summarised in Fig 6.

A limitation of this study is that we have assumed that the mechanism of action of ART26.12 is through FABP5 due to its high selectivity over other FABPs, and the lack of physiologically relevant off-target activity observed in the secondary pharmacology screen including serotonin and dopamine receptors, among others. Previous studies have shown that the analgesic effects of other FABP inhibitors of this series correlate with their activity at FABP5,^26^ and it has been reported that this is lost in FABP5 KO mice.^27^ Nevertheless, further work is needed to confirm that FABP5 inhibition is the primary pharmacodynamic effect underlying ART26.12 OIPN amelioration.

## Conclusions

In conclusion, the potent and selective FABP5 inhibitor ART26.12 appears to be safe and orally active for the treatment and prevention of OIPN in a rat model, with a mechanism of action that involves endocannabinoid targets (predominantly CB_1_, with potential involvement of CB_2_, PPARα, and TRPV1) and lipid modulation. ART26.12 is a promising candidate for the treatment and prevention of OIPN. Regulatory enabling studies with ART26.12 are currently ongoing with the aim of obtaining authorisation to initiate first-in-human studies for the treatment of painful neuropathies.

## Figures and Tables

**Table 1.** Human Phenotype Ontology: Proteins implicated in Axonal Peripheral Neuropathy

Protein	Name	Fold-change	Peripheral axonal neuropathy
CCT5	T-complex protein 1 subunit theta	2.8	Point mutation causes mutilating peripheral sensory neuropathy
KARS1	Lysine tRNA Ligase	1.5	Point mutation causes Charcot Marie Tooth disease
WDR48	WD repeat-containing protein 48	1.5	Mutations can cause Charcot Marie Tooth disease
MARS1	Methionine tRNA Ligase	1.6	Recessive missense mutation causes Charcot-Marie-Tooth disease Phenotype intermediate between demyelinating & peripheral axonal degeneration.
VCP	Valosin-containing protein	1.6	In-frame deletion causes peripheral axonal neuropathy & hypertonia.

NOTE. 5 proteins significantly impacted by 25 mg/kg ART26.12 (*P* < *.05) that are implicated in axonal peripheral neuropathies. Revealed by enrichment analysis against the HP database (*P* = .0095)*.

Abbreviation: VCP, Valosin-containing protein.
